# Clinical characteristics of older patients with COVID-19: a systematic review of case reports

**DOI:** 10.1590/1980-57642021dn15-010001

**Published:** 2021

**Authors:** Luisser Dainner Saavedra Córdova, Alexander Pieter Mayor Vega, Elmer Luján-Carpio, José Francisco Parodi, Enrique Moncada-Mapelli, Isai Armacanqui-Valencia, Jhonatanael Salvador-Ruiz, Dalia Pawer-Pucurimay, Erickson Ydrogo-Cruz, Mylenka Jennifer Chevarría-Arriaga, Macarena Ganoza-Farro, Araceli Meza-Romero, Cynthia Alejandra Zegarra-Rodríguez, Pedro Gustavo Albán-Murguia, Zaira Bailón-Valdez, Naheilli Palacios-Garcia, Danae Quevedo-La-Torre, Angelica Lizeth Alcós-Mamani, Luisa Alisson Gómez-Martel, Max Antonio Roca-Moscoso, Martin Gamboa-Orozco, Alberto Salazar-Granara

**Affiliations:** 1 Universidad de San Martín de Porres, School of Medicine – Lima, Peru.; 2 Research Centre of Traditional Medicine and Pharmacology, School of Medicine, Universidad de San Martín de Porres – Lima, Peru.; 3 Extracurricular Research Internship Program, Research Centre of Traditional Medicine and Pharmacoly, School of Medicine, Universidad de San Martín de Porres – Lima, Peru.; 4 Research Centre of Aging, School of Medicine, Universidad de San Martín de Porres. – Lima, Peru.; 5 Scientific Society of Medical Students, Universidad de San Martín de Porres – Lima, Peru.; 6 Scientific Society of Medical Students, Universidad Pedro Ruiz Gallo – Lambayeque, Peru.; 7 Scientific Association of Medical Students, Universidad del Altiplano – Puno, Peru.; 8 Sociedad Peruana de Farmacología y Terapéutica Experimental – Lima, Peru.

**Keywords:** coronavirus infections, SARS-CoV-2, aged, case reports, systematic review, infecção por SARS-CoV-2, covid-19, idosos, relatos de casos, revisão sistemática

## Abstract

In the context of the current COVID-19 pandemic, higher morbidity and mortality have been reported in older adults. This age group presents physiological changes and its own clinical conditions such as frailty, dementia, among others.

**Objective:**

To describe the characteristics of COVID-19 patients, both over and under 80 years old, by conducting a systematic review of the literature describing case reports, and to summarize and critically assess these characteristics.

**Methods:**

Systematic review. The study was registered on the Registry of Health Research Projects (PRISA) of the Peruvian National Institute of Health (code EI00000631). Five electronic databases (Scopus, PubMed, PubMed Central, LILACS, and SCIELO) were systematically searched during the period between December 31, 2019 and April 16, 2020. The search focused on case reports, case studies, and case series of older people with COVID-19 infection aged over or under 80 years. When selecting the cases, priority was given to clinical and epidemiological profile, laboratory and imaging patterns, and comprehensive geriatric evaluation.

**Results:**

1,149 articles were identified; after applying the filters, a total of 15 publications of case reports and complete records of 27 older adults were obtained. The most frequent age group was between 60 to 69 years old. There is little literature regarding case reports of older adults aged over 80 years. The most frequent parameters were hypertension, fever, cough, respiratory distress, ground-glass opacification in chest radiography and tomography. Furthermore, decrease in PaO_2_/FiO_2_ ratio and lymphocytes, and increase in C-reactive protein and Interleukin 6 were observed.

**Conclusions:**

This systematic review found little available information of patients under 80 years old, and far less for those over 80 years old, and an absence of comprehensive geriatric assessment.

## RESUMO.

No contexto da atual pandemia de covid-19, maior morbidade e mortalidade têm sido relatadas em idosos. Sabe-se que essa faixa etária apresenta alterações fisiológicas e condições clínicas próprias, como fragilidade, demência, entre outras.

**Objetivo:**

Descrever as características de pacientes com covid-19, maiores e menores de 80 anos, por meio de uma revisão sistemática da literatura que descreve relatos de casos, e resumir e avaliar criticamente essas características.

**Método:**

Revisão sistemática. O estudo foi registrado no Registro de Projetos de Pesquisa em Saúde (PRISA) do Instituto Nacional de Saúde do Peru (código EI00000631). Local: cinco bases de dados eletrônicas (Scopus, PubMed, PubMed Central, LILACS e SCIELO) foram sistematicamente pesquisadas entre 31 de dezembro de 2019 e 16 de abril de 2020. A busca se concentrou em relatos de caso, estudos de caso e séries de casos mais antigos pessoas com infecção por SARS-CoV-2 com mais e menos de 80 anos. Na seleção dos casos, a prioridade foi dada ao perfil clínico e epidemiológico, padrões laboratoriais e de imagem, e avaliação geriátrica abrangente.

**Resultados:**

Foram identificados 1.149 artigos. Após a aplicação dos filtros, obteve-se um total de 15 publicações de relatos de caso e registros completos de 27 idosos. A faixa etária mais frequente foi de 60 a 69 anos. Há pouca literatura sobre relatos de casos de adultos com mais de 80 anos. Os parâmetros mais frequentes foram hipertensão, febre, tosse, dificuldade respiratória, vidro fosco na radiografia e tomografia de tórax. Também foram observados diminuição da PaO2 / FiO2 e linfócitos, e aumento da proteína C reativa e Interleucina 6.

**Conclusões:**

Esta revisão sistemática encontrou poucas informações disponíveis sobre pacientes com menos de 80 anos, em quantidade ainda menor para aqueles com mais de 80 anos, além de uma ausência de avaliação geriátrica abrangente.

## INTRODUCTION

On December 31, 2019, a cluster of new pneumonia cases (fever, dry cough, and dyspnea) was reported in the city of Wuhan, China. The outbreak of a new coronavirus (CoV), called Coronavirus Disease 2019 (COVID-19), was thus caused by Severe Acute Respiratory Syndrome Coronavirus 2 (SARS-CoV-2). The infection progressively spread throughout the world.^1^ The World Health Organization (WHO) declared COVID-19 a global pandemic on March 11, 2020, by which date over 5,406,282 cases and 343,562 deaths^2^ had been confirmed.

In Latin America, the first COVID-19 case was reported in Brazil on February 26, 2020,^3^ whereas in Peru the first case was reported on March 6, 2020.^4^ As of the date of this study, Peru has approximately 129,751 confirmed cases and 3,788 deaths.^5^ COVID-19 may affect people of any age; however, with a fatality rate above 8%, people over the age of 70 years with or without comorbidities seem to be more susceptible.^6^ All countries report that the highest mortality rate for severe disease is verified for those aged over 80 years.^7^

One of the factors of greater virulence and severity of COVID-19 in older people are the physiological changes that occur at this age. Immunosenescence is characterized by a decreased function of innate and acquired immunity that may be conducive to an imbalance and, hence, to a chronic proinflammatory state, which make patients susceptible to infections and noncommunicable chronic diseases.^8^

In addition, older people present other clinical conditions, such as frailty, sarcopenia, disability, cognitive decline, anxiety, depression, and others, which exacerbate and are conducive to a negative progression of the disease.^9^ For this reason, researching and classifying these conditions are accepted as the best strategy to define the need for care and to predict the prognosis.^10^ ^,^ ^11^ On the other hand, healthcare systems are not adapted to treat older adults.^12^

Although an individual is considered old from the age of 60 years, various classifications based on age groups have been proposed. However, taking this into consideration, the cutoff at the age of 80 years becomes relevant and is consistent with progressive physiological changes and clinical conditions.^13^

In contrast, in the general population, the current diagnosis of COVID-19 is based on epidemiology history, clinical pattern observation, auxiliary exams, such as chest X-ray and Computed Tomography, and is confirmed by molecular and/or serological tests.^14^ ^–^ ^17^

According to the National Institute of Statistics and Information, older adults in Peru represented 10.14% of the total population in 2017.^18^ The current COVID-19 pandemic is more lethal in older people; in Peru, reports show that 2,609 of the 3,788 deaths were of older adults.^5^ It is necessary to gather information that allows to differentiate older people suffering from COVID-19.

The purpose of this article is to describe the clinical characteristics of COVID-19 patients, aged both over and under 80 years, by conducting a systematic review.

## METHODS

This is an observational, cross-sectional, and systematic review study.

The ethical aspects of the study observed at all times included beneficence, non-maleficence, confidentiality, and justice principles. The study was registered on the Registry of Health Research Projects (PRISA) of the Peruvian National Institute of Health (*Instituto Nacional de Salud –* INS) with code EI00000631.

The systematic review focused on all studies on clinical case reports that presented detailed clinical information of older people with confirmed diagnosis of COVID-19 infection, particularly centered on these four fundamental items:

- Clinical and epidemiological profile.
- Laboratory Tests Pattern.
- Comprehensive Geriatric Assessment (CGA).
- Aged 80 years and over/under 80 years.
- The information used in this study was collected on a step-by-step approach as follows:
- Publication period: studies carried out from December 31, 2019 to April 16, 2020 were considered.
- Study characteristics: case reports, case studies, and case series were used.
- Databases: the databases Scopus, PubMed (Medline), PubMed Central, LILACS (Latin American & Caribbean Health Sciences Literature), and SCIELO (Scientific Electronic Library Online) were consulted.
- Process to identify data acquisition: different Boolean expressions (AND and OR) were used with the descriptive Medical Subject Headings: “COVID-19”, “SARS-CoV-2,” “2019n-CoV,” “new coronavirus,” “novel coronavirus,” “2019-novel coronavirus,” “COVID-19 pandemic,” “COVID-19 virus disease,” “Wuhan coronavirus,” “2019-nCoV disease,” “2019-nCoV infection,” “coronavirus disease-19,” “COVID-19 virus infection,” “aged,” “aged, 80 and over,” “frail elderly,” “oldest old,” “octogenarian,” “nonagenarian,” “centenarian,” “elderly.” The complete search strategy is presented in Annex 1. Next, the titles were reviewed, and duplicate articles were eliminated. Then, the four fundamental items were examined.

Inclusion criteria:

- English, Spanish or Portuguese languages.
- Older adults (aged over 60 years).
- Patients with confirmed diagnosis of COVID-19.
- Complete clinical information.

Exclusion criteria:

- Original articles.
- Review.
- Editorial.
- Short communications.
- Letters.
- Special announcements.
- Any form opposite to the inclusion criteria.

Process for the selection of clinical case reports: seven independent reviewers selected the clinical case reports, in the following order:

- Review of titles and abstract.
- Contrasting of selected studies, and resolution of divergences through discussion and consensus.
- Extensive reading of the clinical case reports.
- Confirmation of clinical case reports after contrasting with the inclusion criteria.

Extraction of objective information and data management: information was independently extracted by seven reviewers; an *ad hoc* data collection form was used. The forms were compared and resolutions were reached through discussion and consensus.

Data presentation: descriptive tables were prepared considering the four fundamental items of the study. Absolute and relative frequencies, mean and standard deviation, median and interquartile range are presented, as appropriate. The *Statistical Package for the Social Sciences* (SPSS) software, version 25, was used.

## RESULTS

Table 1 shows the used keywords and the strategies implemented for the systematic search in the selected databases.

**Table 1:** Keywords and implemented strategies.

Systematic review items
Keywords*
*COVID-19, SARS-CoV-2, Wuhan coronavirus, 2019-nCoV, novel coronavirus, new coronavirus, 2019-novel coronavirus, COVID-19 pandemic, COVID-19 virus infection, coronavirus-19 disease, 2019-nCoV infection, 2019-nCoV disease, COVID-19 virus disease, Aged 80 and over, Oldest-Old, Nonagenarian, Octogenarian, Centenarian, Nonagenarians, Octogenarians, Centenarians, Frail Elderly, Aged, Elderly.* *Anciano de 80 o más Años, Ancianos de 80 Años y más Anciano, Anciano Frágil, Centenarios, Nonagenarios, Octogenarios, Adulto Mayor, Ancianos, Persona Mayor, Persona de Edad, Personas Mayores, Salud de la Persona Anciana, Salud de la Persona Mayor, Salud de la Tercera Edad, Tercera edad, y Longevos.*
SCOPUS search strategy
*(TITLE-ABS-KEY (“COVID-19”) OR TITLE-ABS-KEY (“SARS-CoV-2”) OR TITLE-ABS-KEY (“Wuhan coronavirus”) OR TITLE-ABS-KEY (“2019-nCoV”) OR TITLE-ABS-KEY (“novel coronavirus”) OR TITLE-ABS-KEY (“new coronavirus”) OR TITLE-ABS-KEY (“2019-novel coronavirus”) OR TITLE-ABS-KEY (“COVID-19 pandemic”) OR TITLE-ABS-KEY (“COVID-19 virus infection”) OR TITLE-ABS-KEY (“coronavirus disease-19”) OR TITLE-ABS-KEY (“2019-nCoV infection”) OR TITLE-ABS-KEY (“2019-nCoV disease”) OR TITLE-ABS-KEY (“COVID-19 virus disease”)) AND (TITLE-ABS-KEY (“Aged, 80 and over”) OR TITLE-ABS-KEY (“Oldest Old”) OR TITLE-ABS-KEY (“Nonagenarian”) OR TITLE-ABS-KEY (“Octogenarian”) OR TITLE-ABS-KEY (“Centenarian”) OR TITLE-ABS-KEY (“Nonagenarians”) OR TITLE-ABS-KEY (“Octogenarians”) OR TITLE-ABS-KEY (“Centenarians”) OR TITLE-ABS-KEY (“Frail Elderly”) OR TITLE-ABS-KEY (“Aged”) OR TITLE-ABS-KEY (“Elderly”))*
PubMed Central search strategy
*((((((“Aged, 80 and over”[MeSH Terms] OR (“aged”[MeSH Terms] OR “Frail Elderly”[MeSH Terms])) OR (((((((((“Aged, 80 and over”[Abstract]) OR Aged[Abstract]) OR “Frail Elderly”[Abstract]) OR “Oldest Old”[Abstract]) OR Nonagenarians[Abstract]) OR Nonagenarian[Abstract]) OR Octogenarians[Abstract]) OR Octogenarian[Abstract]) OR Centenarians[Abstract]) OR Centenarian[Abstract]) OR Elderly[Abstract])) OR (((((((((“Aged, 80 and over”[Title]) OR Aged[Title]) OR “Frail Elderly”[Title]) OR “Oldest Old”[Title]) OR Nonagenarians[Title]) OR Nonagenarian[Title]) OR Octogenarians[Title]) OR Octogenarian[Title]) OR Centenarians[Title]) OR Centenarian[Title]) OR Elderly[Title]))))) AND (((COVID-19[Supplementary Concept]) OR (((((((((((((2019-nCoV[Abstract]) OR COVID-19[Abstract]) OR SARS-CoV-2[Abstract]) OR “new coronavirus”[Abstract]) OR “novel coronavirus”[Abstract]) OR “2019-novel coronavirus”[Abstract]) OR “COVID-19 pandemic”[Abstract]) OR “COVID-19 virus infection”[Abstract]) OR “coronavirus disease-19”[Abstract]) OR “2019-nCoV infection”[Abstract]) OR “2019-nCoV disease”[Abstract]) OR “Wuhan coronavirus”[Abstract]) OR “COVID-19 virus disease”[Abstract])) OR (((((((((((((2019-nCoV[Title]) OR COVID-19[Title]) OR SARS-CoV-2[Title]) OR “new coronavirus”[Title]) OR “novel coronavirus”[Title]) OR “2019-novel coronavirus”[Title]) OR “COVID-19 pandemic”[Title]) OR “COVID-19 virus disease”[Title]) OR “Wuhan coronavirus”[Title]) OR “2019-nCoV disease”[Title]) OR “2019-nCoV infection”[Title]) OR “coronavirus disease-19”[Title]) OR “COVID-19 virus infection”[Title]))*
PubMed (Medline) search strategy
*(((((((((((((((((2019-nCoV[Other Term]) OR COVID-19[Other Term]) OR SARS-CoV-2[Other Term]) OR “new coronavirus”[Other Term]) OR “novel coronavirus”[Other Term]) OR “2019-novel coronavirus”[Other Term]) OR “COVID-19 pandemic”[Other Term]) OR “COVID-19 virus disease”[Other Term]) OR “Wuhan coronavirus”[Other Term]) OR “2019-nCoV disease”[Other Term]) OR “2019-nCoV infection”[Other Term]) OR “coronavirus disease-19”[Other Term]) OR “COVID-19 virus infection”[Other Term])) OR COVID-19[Supplementary Concept]) OR (((((((((((((2019-nCoV[Title/Abstract]) OR COVID-19[Title/Abstract]) OR SARS-CoV-2[Title/Abstract]) OR “new coronavirus”[Title/Abstract]) OR “novel coronavirus”[Title/Abstract]) OR “2019-novel coronavirus”[Title/Abstract]) OR “COVID-19 pandemic”[Title/Abstract]) OR “Wuhan coronavirus”[Title/Abstract]) OR “COVID-19 virus disease”[Title/Abstract]) OR “2019-nCoV disease”[Title/Abstract]) OR “2019-nCoV infection”[Title/Abstract]) OR “coronavirus disease-19”[Title/Abstract]) OR “COVID-19 virus infection”[Title/Abstract]))) AND ((((((((((((((((“Aged, 80 and over”[Title/Abstract] OR Aged[Title/Abstract]) OR “Frail Elderly”[Title/Abstract]) OR “Oldest Old”[Title/Abstract]) OR Nonagenarians[Title/Abstract]) OR Nonagenarian[Title/Abstract]) OR Octogenarians[Title/Abstract]) OR Octogenarian[Title/Abstract]) OR Centenarians[Title/Abstract]) OR Centenarian[Title/Abstract]) OR Elderly[Title/Abstract]) OR ((“Aged, 80 and over”[MeSH Terms] OR “aged”[MeSH Terms]) OR “Frail Elderly”[MeSH Terms])) OR ((((((((((“Aged, 80 and over”[Other Term] OR Aged[Other Term]) OR “Frail Elderly”[Other Term]) OR “Oldest Old”[Other Term]) OR Nonagenarians[Other Term]) OR Nonagenarian[Other Term]) OR Octogenarians[Other Term]) OR Octogenarian[Other Term]) OR Centenarians[Other Term]) OR Centenarian[Other Term]) OR Elderly[Other Term])))*
SCIELO search strategy
*(((ab:(2019-nCoV)) OR (ab:(COVID-19)) OR (ab:(SARS-CoV-2)) OR (ab:(“Wuhan coronavirus”)) OR (ti:(2019-nCoV)) OR (ti:(COVID-19)) OR (ti:(SARS-CoV-2)) OR (ti:(“Wuhan coronavirus”)))) AND (((ab:(“Anciano de 80 o más Años”)) OR (ab:(Anciano)) OR (ab:(“Anciano Frágil”)) OR (ab:(“Ancianos de 80 Años o más”)) OR (ab:(“Ancianos de 80 Años y más”)) OR (ab:(“Ancianos de 80 o más Años”)) OR (ab:(Centenarios)) OR (ab:(Nonagenarios)) OR (ab:(Octogenarios)) OR (ab:(“Adulto Mayor”)) OR (ab:(Ancianos)) OR (ab:(“Persona Mayor”)) OR (ab:(“Persona de Edad”)) OR (ab:(“Personas Mayores”)) OR (ab:(“Personas de Edad”)) OR (ab:(“Salud de la Persona Anciana”)) OR (ab:(“Salud de la Persona Mayor”)) OR (ab:(“Salud de la Tercera Edad”)) OR (ab:(“Tercera edad”)) OR (ab:(Longevos)) OR (ti:(“Anciano de 80 o más Años”)) OR (ti:(Anciano)) OR (ti:(“Anciano Frágil”)) OR (ti:(“Ancianos de 80 Años o más”)) OR (ti:(“Ancianos de 80 Años y más”)) OR (ti:(“Ancianos de 80 o más Años”)) OR (ti:(Centenarios)) OR (ti:(Nonagenarios)) OR (ti:(Octogenarios)) OR (ti:(“Adulto Mayor”)) OR (ti:(Ancianos)) OR (ti:(“Persona Mayor”)) OR (ti:(“Persona de Edad”)) OR (ti:(“Personas Mayores”)) OR (ti:(“Personas de Edad”)) OR (ti:(“Salud de la Persona Anciana”)) OR (ti:(“Salud de la Persona Mayor”)) OR (ti:(“Salud de la Tercera Edad”)) OR (ti:(“Tercera edad”)) OR (ti:(Longevos))))*
LILACS search strategy *(af:((tw:(2019-nCoV)) OR (tw:(COVID-19)) OR (tw:(SARS-CoV-2)) OR (tw:(“new coronavirus”)) OR (tw:(“novel coronavirus”)) OR (tw:(“2019-novel coronavirus”)) OR (tw:(““COVID-19 pandemic””)) OR (tw:(““COVID-19 virus infection””)) OR (tw:(““coronavirus disease-19””)) OR (tw:(““2019-nCoV infection””)) OR (tw:(““2019-nCoV disease””)) OR (tw:(““Wuhan coronavirus””)) OR (tw:(““COVID-19 virus disease””)))) AND (af:((af:((tw:(aged)) OR (tw:(“Anciano de 80 o más Años”)) OR (tw:(Anciano)) OR (tw:(“Anciano Frágil”)) OR (tw:(“Ancianos de 80 Años o más”)) OR (tw:(“Ancianos de 80 Años y más”)) OR (tw:(“Ancianos de 80 o más Años”)) OR (tw:(“Centenarios”)) OR (tw:(“Nonagenarios”)) OR (tw:(“Octogenarios”)) OR (tw:(“Viejísimos”)) OR (tw:(““Adulto Mayor””)) OR (tw:(““Ancianos””)) OR (tw:(““Persona Mayor””)) OR (tw:(““Persona de Edad””)) OR (tw:(““Personas Mayores””)) OR (tw:(““Personas de Edad””)) OR (tw:(““Salud de la Persona Anciana””)) OR (tw:(““Salud de la Persona Mayor””)) OR (tw:(“Aged, 80 and over”)) OR (tw:(Aged)) OR (tw:(“Health of the Elderly”)) OR (tw:(“Frail Elderly”)) OR (tw:(“Oldest Old”)) OR (tw:(Nonagenarians)) OR (tw:(Nonagenarian)) OR (tw:(Octogenarians)) OR (tw:(Octogenarian)) OR (tw:(Centenarians)) OR (tw:(Centenarian)) OR (tw:(Elderly)) OR (tw:(““Tercera edad””)) OR (tw:(Longevos)) OR (tw:(“Anciano de 60 o más Años”)) OR (tw:(“Aged, 60 and over”)))) OR (af:((mh:(“Anciano de 80 o más Años”)) OR (mh:(Anciano)) OR (mh:(“Anciano Frágil”))))))*

* Excerpted from Medical Subject Headings of the National Library of Medicine of the United States of America, and Health Sciences Descriptors from BIREME – Latin American and Caribbean Center for Information in Health Sciences. OR and AND are Booleans. SCOPUS: Elsevier's abstract and citation database. PUBMED CENTRAL (PMC) is a free full-text archive of biomedical and life sciences journal literature at the U.S. National Institutes of Health, National Library of Medicine (NIH/NLM). PUBMED (MEDLINE) is a database of references and abstracts on life sciences and biomedical topics of the U.S. National Library of Medicine (NLM). SCIELO: Scientific Electronic Library Online is a bibliographic database supported by the São Paulo Research Foundation (FAPESP) and the Brazilian National Council for Scientific and Technological Development (CNPq). Latin American and the Caribbean Health Sciences Literature (LILACS) is an online bibliographic database in medicine and health sciences, maintained by the Latin American and Caribbean Center on Health Sciences Information.

**Figure 1:**
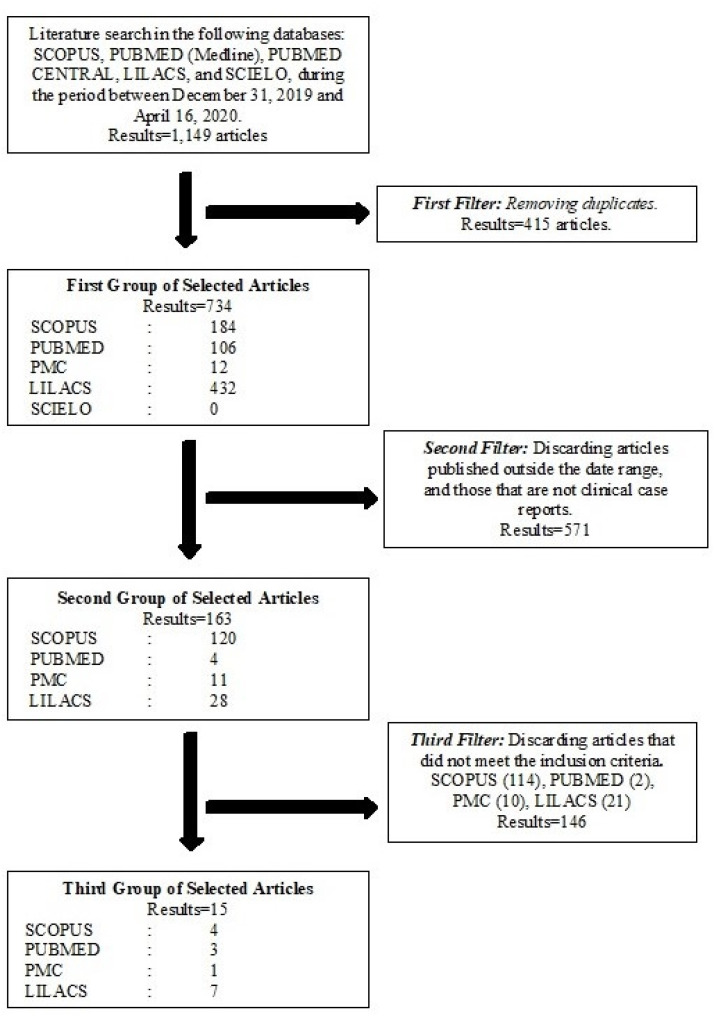
Eligibility screening for systematic review.

The systematic review of the databases yielded a total of 1,149 results. After applying the filters, a total of 15 publications dealing with clinical case reports were obtained, and the review process is shown in Figure 1.

A total of 15 studies that met the inclusion criteria were selected; complete records of 27 older adults were obtained from these clinical case report articles. Table 2 shows epidemiological data, estimated contact times, medical treatment, medical history, signs, and symptoms. Three phases are recorded: outpatient management, hospitalization management, and management in the Intensive Care Unit (ICU). The following frequencies were observed: age group between 60 to 69 years (74.1%), men (51.9%), and residents from Wuhan (63%). The most frequent comorbidity was hypertension (37%); 14% of older people had at least one comorbidity and 6%, more than two comorbidities. The most frequent signs and symptoms were fever, cough, and respiratory distress, which were present in more than 50% of patients.

**Table 2:** Clinical characteristics of older patients with COVID-19 infection.

Features			Total cases	Outpatient management cases	Hospitalization management casesÇ	ICU management cases*
Number			27	3	26	13
Age, median (IQR**)			65 (63–70)	65 (63–66)	65 (63–67.8)	66 (63–69)
Age (%/n)						
	60–69		74.1/20	100/3	76.9/20	76.9/10
	70–79		22.2/6	0	19.2/5	15.4/2
	≥80		3.7/1	0	3.8/1	7.7/1
Sex (%/n)						
	Women		48.1/13	33.33/1	50/13	53.8/7
	Men		51.9/14	66.7/2	50/13	46.2/6
Possible city of infection (%/n)						
	Wuhan-Hubei		63/17	66.7/2	65.4/17	61.5/8
	Other than Wuhan		29.6/8	33.3/1	30.8/8	30.8/4
	No data available		7.4/2	0	3.8/1	7.7/1
Critical times						
	Contact and onset of symptoms	mean±SD	6.97±3.23	–	7.43±2.57	6.56±3.84
		n	15		7	8
	Onset of symptoms and medical evaluation	mean±SD	3.50 (1.25–7.75)€	–	3 (1–4.75)€	5.83±4.63
		n	24		12	12
	Onset of symptoms and hospital admission	mean±SD	6.00 (3.00–8.00)€	–	6.09±5.17	7.08±3.94
		n	23		11	12
	Onset of symptoms and appearance of dyspnea	mean±SD	7±5.29	–	4±6.08	7.75±5.08
		n	15		12	3
Medical history (%/n)						
	Arterial hypertension		37./9	–	–	38.5/5
	Coronary heart disease		3.7/1			0
	Heart failure		3.7/1			0
	Diabetes mellitus		14.8/4	–	–	15.4/2
	Chronic bronchitis	3.7/1	–	–	7.7/1
	Oncological diseaseβ		11.1/3	–	–	7.7/1
	Chronic kidney disease		7.4/2	–	–	7.7/1
	Non-nephrotic proteinuria		3.7/1	–	–	0
	Others^$^		14.8/4			57.1/4
At least one comorbidity		51.8/14		–	–	53.8/7
Two or more comorbidities		22.2/6		33.3/1	19.2/5	7.7/1
Symptoms and Signs (%/n)						
	Fever (%/n)¶		88.9/24	–	92.3/24	85.7/6
	Maximum temperature mean±SD (n)		38 (37.7–39)€	–	38.39±0.6 (9)	36.4±1.20 (2)
Maximum temperature (%/n)						
	≤37.5		18.2/2	0	0	100/2
	37.51–38.3		36.4/4	0	44.4/4	0
	≥38.3		45.5/5	100/3	55.6/5	0
	Cough (%/n)		70.4/19	–	79.2/19	–
	Dry cough		31.6/6	–	–	–
	Not specified		68.4/13	–	–	–
	DyspneaΘ		59.3/16	–	57.7/15	100/13
	Myalgia		25.9/7	–	26.9/7	30.8/4
	Chills		22.2/6	–	23.1/6	30.8/4
	Fatigue		22.6/6	–	23.1/6	0
	Dizziness		7.4/2	–	7.7/2	7.7/1
	Sore throat		7.4/2	–	8.7/2	0
	Diarrhea		7.4/2	–	7.7/2	15.4/2
	Rhinorrhea		3.7/1	–	4.3/1	0
	Other symptoms		22.2/6	–	19.2/5	7.7/1
	Syncope		3.7/1	–	3.8/1	0
	Headache		3.7/1	–	3.8/1	0
	Thoracic oppression		3.7/1	–	3.8/1	0
	Nausea or vomiting		7.4/2	–	3.8/1	7.7/1
	Back pain		3.7/1	–	3.8/1	0
Symptoms leading patients to seek medical care						
	Fever		44.4/12	–	–	46.15/6
	Cough		14.8/4	–	–	7.69/1
	Dyspnea		7.4/2	–	–	15.38/2
	Fatigue		3.7/1	–	–	0
	Diarrhea		3.7/1	–	–	7.69/1
	Syncope		3.7/1	–	–	1
	Dizziness		3.7/1	–	–	7.69/1
	Myalgia		3.7/1	–	–	7.69/1
	Thoracic oppression		3.7/1	–	–	0

Ç in three cases, patients were initially discharged and followed by outpatient management. However, they were subsequently admitted to the hospital. After medical evaluation, 23 other cases were admitted to the hospital.

β three patients had a history of oncological disease: multiple myeloma, thyroid cancer, and gastric cancer.

* Of the 27 cases, one patient was directly admitted to ICU, whereas 12 patients were initially admitted to the hospital and then transferred to ICU (n=3). SD: Standard deviation. IQR: interquartile range.

€ do not present normal distribution according to the statistical test of Shapiro-Wilk (p<0.05), being expressed as median (IQR).

¶ six patients (n=6) who presented fever and chills.

Θ respiratory distress symptoms were assessed in the first medical evaluation and throughout the disease process.

Table 3 shows the results of the imaging tests. In hospitalized patients, the most common finding on radiography was ground-glass opacification (58.3%), lung involvement of bilateral distribution (100%), distribution of peripheral lesions (66.7%), basal opacities (75%), and pleural effusion (58.3%). In contrast, the main findings on tomography were bilateral lung involvement (83.3%) and peripheral location of the lesions (61.1%), considering that the ground-glass opacification pattern (88.9%) prevailed.

**Table 3:** Chest radiography and tomography features in older patients with COVID-19 infection.

Characteristics		Upon admission (n=27)	During hospitalization (n=27)	During ICU stay (n=13)
Chest X-ray performed (%/n)		37/10	44.4/12	57.1/4
Chest X-ray parenchymal radiopacity (%/n)				
	Consolidation	40/4	16.7/2	75/3
	Ground-glass opacification	10/1	58.3/7	25/1
	No radiopacity	30/3	0	0
Affected lung (%/n)				
	Right lung	10/1	0	25/1
	Left lung	10/1	0	0
	Bilateral	40/4	100/12	75/3
Injury distribution (%/n)				
	Peripheral	20/2	66.7/8	0
	Perihilar	10/1	8.3/1	0
	Both	10/1	0	25/1
Injury localization (%/n)				
	Basal opacities	30/3	75/9	50/2
	Apical opacities	0	0	0
	Other locations	10/1	0	25/1
Other findings (%/n)				
	Pleural effusion	0	58.3/7	25/1
	Nodular lesion	0	0	50/2
	Chest CT scan performed (%/n)	37/10	66.7/18	57.1/4
Affected lung (%/n)				
	Right lung	20/2	11.1/2	0
	Left lung	10/1	0	0
	Bilateral	50/5	83.3/15	75/3
Injury localization (%/n)				
	Central injuries	10/1	5.6/1	ND
	Peripheral lesions	20/2	61.1/11	ND
	Both	20/2	5.6/1	ND
Main parenchymal pattern				
	Ground-glass Opacification	70/7	88.9/16	75/3
	Consolidation	10/1	5.6/1	0
	Reticular	0	0	0
	Mixed	0	0	25/1
Other findings (%/n)				
	Nodular lesion	10/1	16.7/3	50/2
	Thickened interlobular	20/2	22.2/4	50/2
	Nonspecific injury margin	ND	11.1/2	ND
	Crazy-paving pattern	30/3	16.7/3	50/2
	Cystic lesion	ND	ND	25/1
	Pleural thickening	ND	16.7/3	50/2
	Pleural effusion	ND	44.4/8	25/1
	Lymphadenopathy	30/3	16.7/3	50/2

ICU: intensive care unit; ND: no data.

Table 4 shows the patients’ results of laboratory tests during their stay in hospital and ICU. In hospitalization, a decrease in PaO_2_/FiO_2_ ratio (mean of 157.75), lymphocytes (mean of 0.72), and platelets (mean of 137.46) were observed. Conversely, there was an increase in lactate dehydrogenase (mean of 502), C-reactive protein (mean of 58.26), urea (mean of 9.44), and Interleukin 6 (IL-6) (mean of 232.53). Regarding ICU patients, there were more acute effects on PaO_2_/FiO_2_ ratio, leukocytes, lymphocytes, and C-reactive protein.

**Table 4:** Laboratory parameters of older patients with COVID-19 infection.

		Normal values	During hospitalization (n=26)	During ICU stay (n=7)
Laboratory values mean±SD (n)				
	PaO_2_/FiO_2_ ratio	>400	157.75±68.26	81±7.07 (2)
	Hemoglobin (g/dl)	13–17.5	13 (5)	–
	Leukocytes count (*10^9/L)	3.5–9.5	4.61 (4.13–6.73)/17€	17.54±5.87 (3)
	Lymphocytes count (*10^9/L)	1.10–3.20	0.72±0.28 (17)	0.42±0.08 (3)
	Neutrophils count (*10^9/L)	1.8–6.3	3.92±1.46	–
	Platelets count (*10^9/L)	150-450	137.46±37.87	–
	CD4 (cells/μL)	34–52	39.32±10.61	–
	CD8 (cells/μL)	21–39	17.28±5.80	–
	Myoglobin (ng/mL)	0–110	40.10 (32.7–111.9)/7€	–
	Troponin (ngmL)	0–0.1	0.012 (0.012–0.014)/(8)€	–
	Creatine phosphokinase -MB (ng/ml)	0–2.37	0.90 (0.23–42.00)/11€	–
	Lactate dehydrogenase (UI/L)	114.0–240.0	502±310.90 (14)	–
	Glutamic-oxaloacetic transaminase (GOT/AST) (UI/L)	5-40	33.6 (24.6–49.00)/13€	–
	Glutamic pyruvic transaminase (GPT/ALT) (UI/L)	5-40	26.5 (16.50–39.65)/13€	–
	Albumin (g/L)	40.0–55.0	38.28±2.99 (11)	–
	C-reactive protein (mg/L)	<10	58.26±34.42 (16)	208±93.24 (3)
	Creatinine (μmol/L)	58–110	81.90 (53.55–98.50)/13€	–
	Urea (mmol/L)	0.5-2.7	9.44±4.93 (12)	–
	Interleukin 6 (pg/mL)	<1.5	232.53±209.97 (3)	–
	D-dimer (mg/L)	<0.5	0.45 (0.30–0.60)/4€	–
	Procalcitonin (ng/mL)	0–0.5	0.09 (0.04–0.20)/8€	–

€ non-Gaussian distribution according to the Shapiro-Wilk statistical test (p<0.05), in such a way that they are expressed as median and interquartile range (IQR/n); NV: normal values.

Table 5 shows the medications used during hospitalization and ICU. During hospitalization, the most frequent treatment observed consisted in antivirals (51.9%), of which Interferon was the most used (57.1%). Antibiotics and antivirals were used in the ICU in a similar frequency, and the most widely used antiviral was the Lopinavir-Ritonavir combination (75%).

**Table 5:** Medication pattern among older patients with COVID-19 infection at the Intensive Care Unit and at the hospital.

Treatments (%/n)		During hospitalization (n=27)	Destination after hospitalization			During ICU stay (n=13)	Destination after ICU stay		
			Medical discharge	ICU	Not specified		Medical discharge	Death	Stay in ICU
Antibiotics		14.8/4	25/1	50/2	25/1	30.77/4	50/2	25/1	25/1
Antivirals		51.9/14	14.3/2	64.3/9	21.4/3	30.77/4	50/2	25/1	25/1
Lopinavir-Ritonavir		37.5/5	20/1	60/3	20/1	75/3	66.7/2	0	33.3/1
Oseltamivir		28.6/4	0	100/4	0	0	0	0	0
Ribavirin		50/7	0	85.7/6	14.3/1	0	0	0	0
Others		85.7/12	16.7/2	58.3/7	25/3	15.4/2	0	0	0
Interferon		57.1/8	0	75/6	25/2	0	0	0	0
Umifenovir		21.4/3	66.7/2	33.3/1	0	7.7/1	0	0	100/1
Abidor		7.14/1	0	0	100/1	0	0	0	0
Remdesivir		–	–	–	–	7.7/1	0	100/1	0
Antimalarial treatment										
	Hydroxychloroquine	100/2	0	100/2	0	15.4/2	100/2	0	0
	Immunological treatment	14.8/4	50/2	25/1	25/1	23.1/3	66.7/2	0	33.3/1
	Gamma globulin	75/3	33.33/1	33.33/1	33.33/1	7.7/1	0	0	100/1
	Tocilizumab	25/1	100/1	0	0	–				
	Convalescent plasma	–	–	–	–	15.4/2	100/2	0	0
Other treatments									
	Chinese Traditional Medicine	11.1/3	33.33/1	33.33/1	33.33/1	7.7/1	0	0	100/1
	Abidor+”Chinese Traditional Medicine”+Methylprednisolone	33.3/1	0	0	100/1	0	0	0	0
	Lopinavir/Ritonavir/Umifenovir+Shufeng Jiedu (SFJDC)	66.67/2	50/1	50/1	0	100/1	0	0	100/1
	Methylprednisolone	23.1/3	33.33/1	0	33.7/1	15.4/2	100/2	0	0

ICU: intensive care unit.

## DISCUSSION

### Epidemiological characteristics

During the COVID-19 pandemic, older people have disproportionately been severely affected by the disease, and indeed have required hospitalization and accounted for high death rates, particularly those aged over 80 years.^19^ Considering this new virus, it is undoubtedly necessary to collect new data and develop evidence-based strategies concerning prevention and management of people affected by the virus.^20^ This research only found 27 reported cases of older adults with COVID-19 that met the inclusion criteria; their median age was approximately 65 years and only one case was of an individual aged over 80 years.^21^ Despite this group being the most affected by this pandemic, there are no significant studies on epidemiology or clinical manifestations of COVID-19 in older adults.

Likewise, the relatively low median age reported by this investigation would explain the verified evolution: only one death, 3.7% of the total. Wu et al.,^22^ in a cohort of patients, found that aging over 65 years alone is an independent risk factor for developing acute respiratory distress syndrome (ARDS) and for death probably due to less rigorous immune response. These results have been corroborated by various publications worldwide.^23^ ^,^ ^24^

On the other hand, some research in older people with COVID-19 have indicated that the contributing factors for poor health outcomes include the physiological changes of aging,^25^ but mainly the multiple comorbidities related to age,^26^ such as heart and lung diseases, diabetes, dementia, and polypharmacy. However, a preprint has found that, in older adults from Mexico, comorbidities or inequalities in accessing healthcare systems (difficulty in accessing a healthcare service or not having health insurance) are predictors of severity for COVID-19, regardless age.^27^ Hence, it should be noted that some literature has reported that a significant proportion of older people living in nursing homes failed to be hospitalized and died, drawing attention to their limited access to healthcare services such as an adequate management with a comprehensive geriatric approach, including palliative care.^19^ ^,^ ^28^ ^–^ ^30^

Moreover, health profiles, stress response, and functional ability of older adults significantly vary. While some of them have low intrinsic capacity — i.e., they are care-dependent —, others have high functional capacities and actively participate in and contribute to their communities.^31^ Geriatric medicine has shown that efficient health interventions in older adults should be determined by the conditions of frailty, functional ability, and care dependence of older adults.^32^ ^–^ ^35^ In addition, reporting and generating information on some specific variables, such as polypharmacy, living in long-term care facilities, multiple comorbidities, and physical dependence, would enrich the literature on COVID-19 and older people available to date.^20^ ^,^ ^36^ However, in this investigation, no case report referred to the evaluation of functionality, frailty condition, functional, cognitive and emotional state, or to polypharmacy or regular residence in long-term institutions. All of these aspects are considered important for the prognosis of older people.

Regarding the epidemiological characteristics of older adults with COVID-19, this study, like reports from other countries,^5^ ^,^ ^20^ ^,^ ^31^ ^–^ ^38^ found that men aged 60 to 69 years were the most affected and that more than half of older people with COVID-19 had some comorbidity, and this proportion is much higher when compared with young patients with underlying conditions.^39^ For Liu et al.,^38^ these findings would indicate weaker immune functions in male older adults, thus increasing the risk of infection by SARS-CoV-2.

### Clinical manifestations

Furthermore, a large percentage of older adult patients with COVID-19 included in this review had fever when admitted to the hospital, a fact that has also been reported by other investigations^40^ ^,^ ^41^ in middle-aged groups. Likewise, Zhou et al.^42^ report an average of 11 days lapsing from the onset of symptoms to admission in patients aged 46 to 67 years and an average of seven days from the onset of the first symptom to the appearance of dyspnea. In contrast, this study found that, in this population, an average of six days lapsed from the onset of the first symptom to hospitalization, and an average of seven days until the onset of dyspnea.

An important result of this investigation suggests that dyspnea is not a frequent symptom at hospital admission in adults and older patients with COVID-19, but is rather developed later. This is consistent with the severity of the disease. Previous studies have shown that the duration from symptom onset to dyspnea was 7–8 days in patients with COVID-19,^41^ ^,^ ^42^ one day after hospital admission. Those studies have also shown that only 18.7% of patients have dyspnea at the time of admission,^19^ ^,^ ^40^ ^–^ ^43^ but it was usually more frequent in patients admitted to the ICU, who required mechanical ventilation, or who died.^43^ ^,^ ^44^ Although these investigations focused their study on the adult population, these results are similar to those found in the present review, considering the low median age of older people already discussed. In contrast, the absence of dyspnea at the time of the medical consultation can be explained by Gattinoni et al.,^45^ who indicate that patients infected with SARS-CoV-2 would initially have severe hypoxemia due to vasoplegia, which, on account of low lung compliance, can increase ventilation and compensate it. This phenomenon is called silent hypoxemia.

### Radiological findings

Regarding the imaging findings, it was evident that the most predominant radiological finding at the time of admission, during hospitalization, and in the ICU was the ground-glass opacification pattern, a result similar to that found in other studies.^46^ ^–^ ^51^ However, this investigation only found a few cases with sequential radiological examinations. In this regard, it is worth considering the timing of the X-ray or CT, since there is evidence that the patterns may vary specifically depending on the timing of the natural history of the disease.^47^ Thus, it is suggested that, in future radiological studies, the day of the imaging examination should be considered regarding the patients’ onset of symptoms. Likewise, it was found that the locations of the lesions were mostly peripheral, which is in accordance with what has been reported in younger ages.^47^ ^–^ ^50^ Conversely, Salehi et al.,^48^ found atypical initial imaging presentation of consolidative opacities superimposed on ground glass mainly in older populations. In this review, a basal consolidation pattern was more frequent observed in plain chest X-rays, whereas, the ground-glass pattern is more likely to be observed in CT scan. Therefore, the imaging findings in older people do not differ from that found in younger age groups, and the CT scan, due to its high sensitivity for the diagnosis of COVID-19,^51^ could potentially be used as a detection technique in epidemic areas. Nevertheless, it is necessary to carry out more studies and consider a larger sample to determine this hypothesis.

### Laboratory features

Moreover, alterations in laboratory tests were similar to those presented in other articles for middle-aged groups.^52^ ^,^ ^53^ Concerning altered values, the PaO_2_/FiO_2_ ratio was less than 150 in most of the patients, demonstrating that the infection causes a severe level of hypoxemia.^53^ In the white series, leukocytosis in patients admitted to the ICU was found. In contrast, lymphopenia worsens during the ICU stay, with decrease in the CD8 lymphocyte group. These findings corroborate another study in which decreases in leukocytosis, CD8 lymphocytes, and Natural Killer cells occur, possibly associated with overexpression of the Natural-Killer receptor group 2-A (NKG2A), which is induced by SARS-CoV-2 causing functional fatigue of the lymphocytes from the initial phase of the disease.^54^ ^,^ ^55^ The evaluation of troponins was normal, despite 37% of the patients having some cardiovascular history. A meta-analysis found that troponins were elevated in patients with severe COVID-19.^56^ Finally, the presence of increased urea values is associated with higher hospital mortality in COVID-19 patients due to reported kidney involvement.^57^

It is worth emphasizing that, according to laboratory evidence, the inflammatory process has been present in older people with COVID-19 since hospitalization. According to Zhao et al.,^58^ in a study that included 82.4% older people, the increase in C-reactive protein, interleukin 6, and procalcitonin was observed in more than 60% of cases. Likewise, another study indicated that increased lactate dehydrogenase and C-reactive protein are strongly associated with the presence of severe COVID-19 disease.^59^ Both results are supported by Chen et al.,^43^ who indicate that low values of these markers are associated with patients who recovered from the disease. On the other hand, excessive increase in IL-6 after acute lung damage leads to multiple organ failure (MOF) and to a cytokine storm.^45^ Additionally, increase in C-reactive protein may be partly attributed to the effects of IL-6.^60^ Although the production of procalcitonin in viral infections is inhibited by interferon (INF-y), procalcitonin is elevated in severe patients. This could reflect the presence of an over-aggregated bacterial infection, which would cause increase in the concentrations of interleukin 1 beta (IL1-b), tumor necrosis factor alpha (TNF-a), and IL-6.^61^

### Used therapies

Regarding treatment, antivirals were the most frequently used drugs in older adults, but their efficacy for the treatment of COVID-19 is unclear. This review found that 51.9% of hospitalized older patients with COVID-19 received some antiviral; however, 64% of them ended up in the ICU. One of the most widely used antivirals was Lopinavir-Ritonavir. Nevertheless, the only clinical trial of this drug observed that hospitalized adult patients with severe COVID-19 did not show any benefits over standard care.^62^ The second most widely used antiviral during hospitalization in the reviewed cases was Interferon. Recently, a clinical trial carried out by Hung et al.,^63^ evaluated the triple therapy of Interferon beta-1b, Ribavirin plus Lopinavir-Ritonavir, finding that this combination has better performance than the treatment with Lopinavir-Ritonavir alone in alleviating symptoms and shortening duration of virus clearance and hospital stay in patients with mild to moderate COVID-19 infection. However, this study did not have a placebo group and the triple therapy was only administered to those patients with an illness duration of seven days or less. On the other hand, Remdesivir, an experimental antiviral (GS-5734), has been subject to only one clinical trial to date; such study found that adult patients with a median age of 65 years, admitted to the hospital for severe COVID-19, and given the drug had no clinical benefits.^64^ Nevertheless, the complete results of a clinical trial funded by the US National Institute of Allergy and Infectious Diseases are still being evaluated and, according to its preliminary report, such drug would have shortened the recovery time of hospitalized adults with COVID-19 (average age of 59 years).^65^ Other antivirals, such as Oseltamivir, have limited activity against SARS-CoV-2, while Favipiravir and Umifenovir, the internationally available influenza antivirals, have different viral targets and require further investigation.^66^

Likewise, aminoquinolines was not frequently used for the treatment of older people (two out of the 27 cases); however, the latest studies on aminoquinolines are controversial. Initially, Gautret et al.,^67^ found that Hydroxychloroquine treatment was significantly associated with reduced viral load in patients with COVID-19 and its effect was enhanced by Azithromycin. However, this study did not include the criterion of clinical severity of participants, the mean age was around 51.2 years, no randomization was used, and sample size was limited. Then, Tang et al.,^68^ found that Hydroxychloroquine treatment did not result in a significantly higher likelihood of negative conversion than standard care alone of hospitalized adult patients (mean age of 46 years) with mild to moderate COVID-19 infection. Other observational studies found that treatment with Hydroxychloroquine, Azithromycin, or both was not significantly associated with differences in hospital mortality,^69^ risk of intubation, or death,^70^ and it did not appear to have any effect on reducing admissions to intensive care or death.^71^ Furthermore, in patients aged 60 years and older, Hydroxychloroquine for the treatment of COVID-19 has a high risk of prolongation of the corrected QT (QTc) interval, and concurrent treatment with Azithromycin is associated with greater changes in electrocardiogram.^72^ To date, the clinical trial from Mehra et al.,^73^ conducted with 96,032 adult patients (mean age of approximately 53.8 years), the largest sample to date, failed to determine a benefit of Hydroxychloroquine or Chloroquine, when used alone or with a macrolide, in the hospital results of COVID-19; those who did not survive had a mean age of 60 years and had some comorbidity. Thus, taking into account the current evidence, the administration of aminoquinolines in older people should be done with caution and be strictly monitored, considering adverse effects and polypharmacy due to the large number of interactions with other drugs. Even in countries where Hydroxychloroquine has been indicated to treat COVID-19, such use should be reconsidered or the monitoring and care should be strengthened.

As for immunological treatments in older adults with COVID-19, scientific evidence is insufficient to support their use; however, new clinical evidence could shed light on such treatments. To date, there are no published results of clinical trials on convalescent plasma treatment,^74^ except one clinical trial (NCT04345991) that will finish its study in June 2021^75^ and nine others that will be completed in the following months. However, no specification has been given on whether they will assess older people and whether the results of this group will be separately presented. Similarly, there are no clinical trials to support Tocilizumab treatment. However, the relevant intervention of IL-6 in cytokine storm allows some authors to theorize that Tocilizumab may become an effective medicine for patients with severe COVID-19.^46^

An important outcome of the present study was the scarce information found in the literature regarding case reports of COVID-19 infection in adults aged over 80 years.

In addition, the authors found no evidence of case reports of COVID-19 infection in older patients that considered specific conditions, such as frailty, dementia, emotional status, level of functionality, polypharmacy, and others, and how to manage them.

The age group was between 60 to 69 years, and the main clinical, laboratory, radiological, and therapy-related findings were as follows: a) more than one comorbidity; b) fever, which was the most frequently reported manifestation at hospital admission; c) dyspnea, which was the most frequently reported manifestation at hospitalization; d) imaging of ground-glass opacification, which was the main outcome at hospital admission and hospitalization; e) increased level of lactate dehydrogenase, C-reactive protein, interleukin 6, and procalcitonin were the most frequently observed features; and f) antivirals, Hydroxychloroquine, antibiotics, corticoids, monoclonal antibodies, and herbal drugs were used as treatment options.
